# Effects of alendronate on cartilage lesions and micro-architecture deterioration of subchondral bone in patellofemoral osteoarthritic ovariectomized rats with patella-baja

**DOI:** 10.1186/s13018-024-04677-0

**Published:** 2024-03-25

**Authors:** Mingjian Bei, Zhiyuan Zheng, Yaping Xiao, Ning Liu, Xuehui Cao, Faming Tian, Liu Zhang, Xinbao Wu

**Affiliations:** 1 Department of Orthopedic Surgery, Beijing Jishuitan Hospital Affiliated to Capital Medical University, Xinjiekoudongjie 31, Xicheng Dis, Beijing, 100035 People’s Republic of China; 2 School of Public Health, North China University of Science and Technology, Tangshan, Hebei People’s Republic of China; 3 The Department of Orthopedic Surgery, Wuhan Third Hospital, Tongren Hospital of Wuhan University, No. 241, Pengliuyang Road, Wuhan, 430000 People’s Republic of China; 4 Department of Orthopedic Surgery, Emergency General Hospital, Xibahenanli 29, Chaoyang District, Beijing, 100028 People’s Republic of China

**Keywords:** Alendronate, Patellofemoral osteoarthritis, Patella baja, Animal model

## Abstract

**Background:**

Patellofemoral osteoarthritis (PFJOA) is a subtype of knee OA, which is one of the main causes of anterior knee pain. The current study found an increased prevalence of OA in postmenopausal women, called postmenopausal OA. Therefore, we designed the ovariectomized rat model of patella baja-induced PFJOA. Alendronate (ALN) inhibits osteoclast-mediated bone loss, and has been reported the favorable result of a potential intervention option of OA treatment. However, the potential effects of ALN treatment on PFJOA in the ovariectomized rat model are unknown and need further investigation prior to exploration in the clinical research setting. In this study, the effects of ALN on articular cartilage degradation and subchondral bone microstructure were assessed in the ovariectomized PFJOA rat model for 10 weeks.

**Methods:**

Patella baja and estrogen withdrawal were induced by patellar ligament shortening (PLS) and bilateral ovariectmomy surgeries in 3-month-old female Sprague–Dawley rats, respectively. Rats were randomly divided into five groups (n = 8): Sham + V; OVX + V, Sham + PLS + V, OVX + PLS + V, OVX + PLS + ALN (ALN: 70 μg/kg/week). Radiography was performed to evaluate patellar height ratios, and the progression of PFJOA was assessed by macroscopic and microscopic analyses, immunohistochemistry and micro-computed tomography (micro-CT).

**Results:**

Our results found that the patella baja model prepared by PLS can successfully cause degeneration of articular cartilage and subchondral bone, resulting in changes of PFJOA. OVX caused a decrease in estrogen levels in rats, which aggravated the joint degeneration caused by PFJOA. Early application of ALN can delay the degenerative changes of articular cartilage and subchondral bone microstructure in castrated PFJOA rat to a certain extent, improve and maintain the micrometabolism and structural changes of cartilage and subchondral bone.

**Conclusion:**

The early application of ALN can delay the destruction of articular cartilage and subchondral bone microstructure in castrated PFJOA rat to a certain extent.

**Supplementary Information:**

The online version contains supplementary material available at 10.1186/s13018-024-04677-0.

## Introduction

Osteoarthritis (OA) is a degenerative form of arthritis that affects more than 300 million people worldwide at the hip and knee joints alone [1]. OA is a progressive joint degeneration characterized by synovial inflammation, articular cartilage degeneration, deterioration of subchondral bone (SB) and osteophyte formation. Pain and disability due to knee OA are more strongly associated with the patellofemoral joint (PFJ) than the tibiofemoral joint, while the PFJ is easily overlooked clinically [2]. PFJ OA is a subtype of knee OA, has a high incidence of 24% females and 11% males over the age of 55 years [3]. Biomechanical changes, such as patella baja, trochlea dysplasia, patellofemoral joint instability, etc., are involved in the occurrence and development of OA. Symptoms can be alleviated by activity modification, physical therapy, drugs, weight reduction, McConnell taping, bracing, and injection therapy [4]. Surgical treatment consisted of joint preservation and patellofemoral replacement with the goal of optimizing load distribution and improving patellar alignment and trajectory. Unfortunately, none of these procedures produced definitive long-term outcomes [5].

Epidemiological evidence found an incremental prevalence of OA in postmenopausal women [1], and it has been entitled as the postmenopausal OA in several studies [6, 7]. Estrogen plays an important role in the maintenance of bone mass, the alleviation of cartilage degeneration and the maintenance of systemic bone homeostasis by binding to estrogen receptors in articular cartilage, SB and synovium [8]. Inflammation, aging, and apoptosis of chondrocytes induced by estrogen deficiency exacerbate extracellular matrix degradation. In addition, the abnormal remodeling of SB induced by estrogen deficiency is also involved in the occurrence of OA, which is characterized by a high rate of bone turnover and destruction of SB microstructure, which in turn aggravates cartilage destruction.

Alendronate (ALN), an third-generation bisphosphonates, has been approved for the treatment of osteoporosis due to their ability to inhibit osteoclast-mediated bone loss [9]. Clinical and animal studies have reported favorable results of bisphosphonates as a potential intervention option of OA treatment. ALN has been reported to be effective in reducing osteophyte formation [10, 11] and cartilage degeneration [12] in OA. Moreover, ALN blocks OA matrix metalloproteinase-13 (MMP-13) expression and increases collagen type II (Col-II) expression to reduce OA extracellular matrix degeneration [13]. Taken together, ALN may have therapeutic effects on OA. However, little is known about the specific efficacy of ALN in postmenopausal patients with PFJOA. Therefore, this study aimed to evaluate the role of ALN on the progression of PFJOA in an ovariectomized (OVX) rat model.

## Methods

### Animal handling

A total of 40 three-month-old female Sprague–Dawley rats (253 ± 15.58 g) (Vital River Experimental Animal Technical Co., Ltd., Beijing, China) were used in this study. All rats were housed in standard conditions (22ºC with a 12-h lights on/off cycle) and allowed unlimited food and water. Patella baja and estrogen withdrawal were induced by patellar ligament shortening (PLS) and bilateral ovariectomy surgeries [14], respectively. Briefly, The PLS surgery was conducted under general anesthesia in a sterile environment, with the skin of the right knee shaved and sterilized. Firstly, a 1 cm longitudinal incision was made medially along the patella to the tibial tuberosity. The patellar tendon was then carefully detached, avoiding exposure of the joint cavity. Secondely, a Kirschner wire (7 mm long, 2 mm diameter) with 1-0 nylon sutures in a groove 1 mm medial to both ends was inserted under the patellar tendon from the medial to the lateral region. The sutures were crossed at the proximal end of the tendon. Thirdly, the sutures were threaded under both grooves and tightly tied around the patellar tendon with the knee in its straightest position. Finally, the skin was sealed using 3–0 nylon sutures (Additional file 1: Fig. S1). Rats were injected subcutaneously either ALN (70 μg/kg/week) or vehicle at 72 h post-surgery for 10 weeks in the following groups (n = 8): Sham + V; OVX + V, Sham + PLS + V, OVX + PLS + V, OVX + PLS + ALN. Body weights were measured before surgery and recorded weekly, and drug doses were adjusted accordingly. All rats were killed at 10 weeks and the study was approved by the Institutional Animal Care and Use Committee.

### Radiography

The patellar height ratio was evaluated by X-rayed at 10 weeks post-surgery. All rat’s right knee joint with approximately 90° flexion by a “specific devices”. The lateral position detection under the X-radiography system (settings: 75 kV, 50 Hz, Seriate (BG) Italy, Seriate (BG) Italy) was performed according to the Insall-Salvati (IS) ratio. All imaging analysis were blindly performed by 3 independent researchers based on previous study [15].

### Macroscopic images and analysis

All samples for macroscopic scoring were recorded using a digital camera (Canon 550D; Canon, Japan). Samples were fixed in 100% ethanol for micro-CT analysis. The severity of gross lesions was defined using a scoring system described by Guingamp et al. [16] (Table 1).

**Table 1:** Grading system for macroscopic lesions of cartilage

Grade	Cartilage
0	Normal appearance
1	Slight yellowish discoloration of the chondral surface
2	Small cartilage erosions in load-bearing areas
3	Large erosions extending down to the subchondral bone
4	Large erosions with large areas of subchondral bone exposed

### Micro-CT imaging

To investigate the effects of ALN on micro-architecture deterioration in subchondral bone of patellofemoral joint. Three-dimensional trabecular analysis was performed using a SkyScan1176 (Bruker, Kontich, Belgium). The scanning procedure and region of interest (ROI) of the femoral trochlear were according to our previous study [17]. The densitometry and cancellous microstructure were characterized using CT Analyser and CT vox software (Bruker, Kontich, Belgium) to determine bone mineral density (BMD), bone volume ratio (BV/TV), trabecular separation (Tb.Sp), trabecular pattern factor (Tbpf), trabecular thickness (Tb.Th), trabecular number (Tb.N) and structure model index (SMI).

### Specimen processing and Mankin scoring

After the Micro-CT examination, we immediately fixed all samples used for histological evaluation with 10% neutral buffered formalin. Next, all samples were decalcified in 10% EDTA-Na2 for 10 weeks before embedding in paraffin. Horizontal sections of 6um thickness are prepared for histomorphometry and immunohistochemistry. Sections were stained using toluidine blue for histomorphometry, and cartilage lesions were evaluated with a standard 14-point Mankin scale [18].

### Immunohistochemistry

Immunohistochemical analysis was performed to investigate the changes in molecular mechanism in cartilage degeneration, the following procedures were performed as previous described [13]: Briefly, the deparaffinized sections were rehydrated, digested with 0.05% trypsin for 30 min and incubated for 15 min in 3% H_2_O_2._ After washing with 5% bovine serum albumin in phosphatebuffered saline (PBS), then incubated over night at 4 °C with the following antibodies:Col-II (1:100, II-II6B3, Linsenmayer TF), MMP-13 (1:100, Bioss Inc., Beijing. USA) and Caspase-3 (1:100, Boster Co.Ltd., Wuhan, China). The remaining procedures were performed according to the instructions provided by the PV-6000 Polink-1 HRP DAB Detection System (ZSGB-BIO Corp., China) and the ZLI-9018 DAB kit (ZSGB-BIO Corp., China). Finally, the slides were counterstained with hematoxylin. The image collection (200 × magnification) and result analysis (IOD/mm^2^) were done using a BX53 microscope system (Olympus, Tokyo, Japan) and Image-Pro Plus 6.0 software (Media Cybernetics, Inc, US), respectively.

### Statistical analysis

All data were analyzed using SPSS version 19.0 (SPSS, Chicago, IL, USA). The macroscopic and histological data were presented as the Mean with a 95% confidence interval (CI), other data were presented as mean ± standard deviation. Non-Gaussian distributed data were analyzed using Kruskal–Wallis and Mann–Whitney analyses. Additionally, the Gaussian distributed data were analyzed using a one-way ANOVA followed by Fisher’s least significant difference (LSD) t-test or Dunnett’s T3 test. The *P* value < 0.05 was considered statistically significant.

## Results

### Radiographic findings

**Fig. 1:**
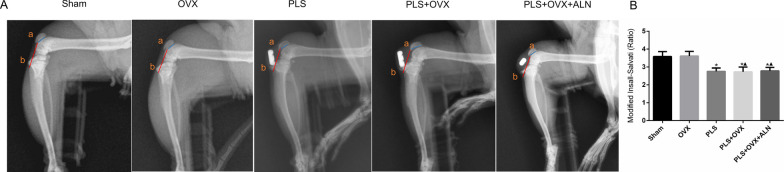
X-ray photography 10 days after molding and modified Insall-Salvati ratio. *p < 0.05 versus sham group, ▲*p* < 0.05 versus OVX group

As shown in Fig. 1, X ray radiography and manual palpation confirmed that the Kirschner wire was fixed in the established position without detachment. Compared with Sham group, Modified Insall-Salvati Ratio of PLS group, PLS + OVX group and PLS + OVX + ALN group was significantly lower than that of Sham group (all *P* < 0.05), which proved that the patella baja model of rats was successfully prepared by PLS operation. There was no significant difference in Modified Insall-Salvati Ratio between Sham group and OVX group (*p* > 0.05).

### Micro-CT findings

**Fig. 2:**
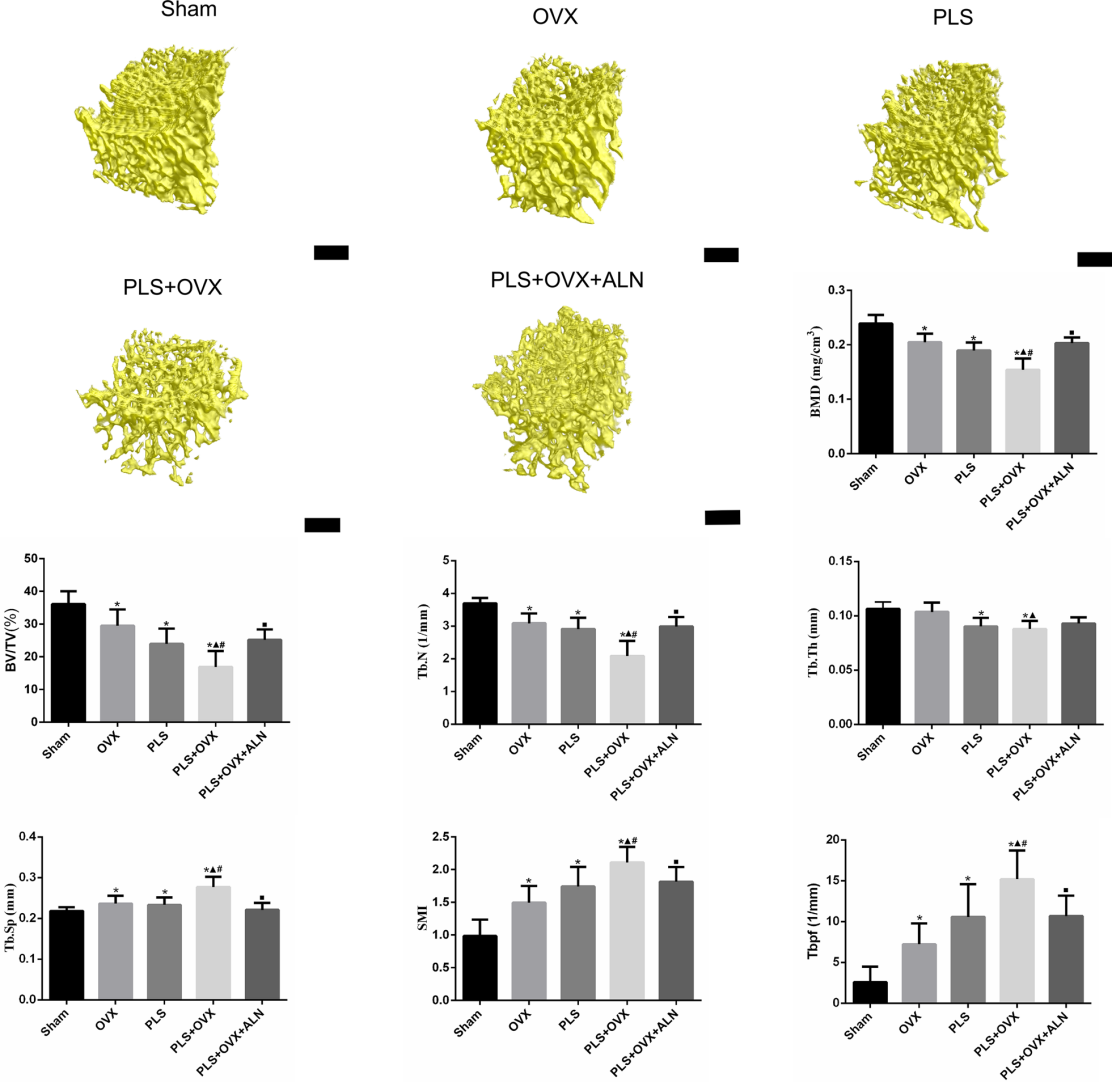
The Micro-CT images and results of the subchondral bone. Black bar = 250 μm. *p < 0.05 versus sham group, ▲*p* < 0.05 versus OVX group, □*p* < 0.05 versus PLS group, ■*p* < 0.05 versus PLS + OVX group

Subchondral bone Micro-CT examination of bone trabecular imaging gross observation showed that the trabeculae of OVX and PLS groups were sparser than those of Sham group (Fig. 2). The trabeculae of PLS + OVX groups were sparser than those of OVX and PLS groups. The trabeculae of PLS + OVX + ALN group were denser than those of PLS + OVX group.

Micro-CT results of subchondral bone (Fig. 2) showed that BMD, BV/TV and Tb.N in OVX group were significantly lower than those in Sham group (all *P* < 0.05), while Tb.Sp, SMI and Tbpf were significantly higher than those in Sham group (all *P* < 0.05). BMD, BV/TV, Tb.N and Tb.Th in PLS group were significantly lower than those in Sham group (all *P* < 0.05), while Tb.Sp, SMI and Tb.pf were significantly higher than those in Sham group (all *P* < 0.05). BMD, BV/TV, Tb.N and Tb.Th in PLS + OVX group were significantly lower than those in Sham group (all *P* < 0.05), while Tb.Sp, SMI and Tbpf in PLS + OVX group were significantly higher than those in Sham group (all *P* < 0.05). Compared with OVX group, BMD, BV/TV, Tb.N and Tb.Th in PLS + OVX group were significantly decreased (all *P* < 0.05), while Tb.Sp, SMI and Tb.pf were significantly increased (all *P* < 0.05). Compared with PLS group, BMD, BV/TV and Tb.N in PLS + OVX group were significantly decreased (all *P* < 0.05), while Tb.Sp, SMI and Tb.pf were significantly increased (all *P* < 0.05). Compared with PLS + OVX group, BMD, BV/TV and Tb.N in PLS + OVX + ALN group were significantly increased (all *P* < 0.05), while Tb.Sp, SMI and Tb.pf were significantly decreased (all *P* < 0.05).

### Macroscopic findings

**Fig. 3:**
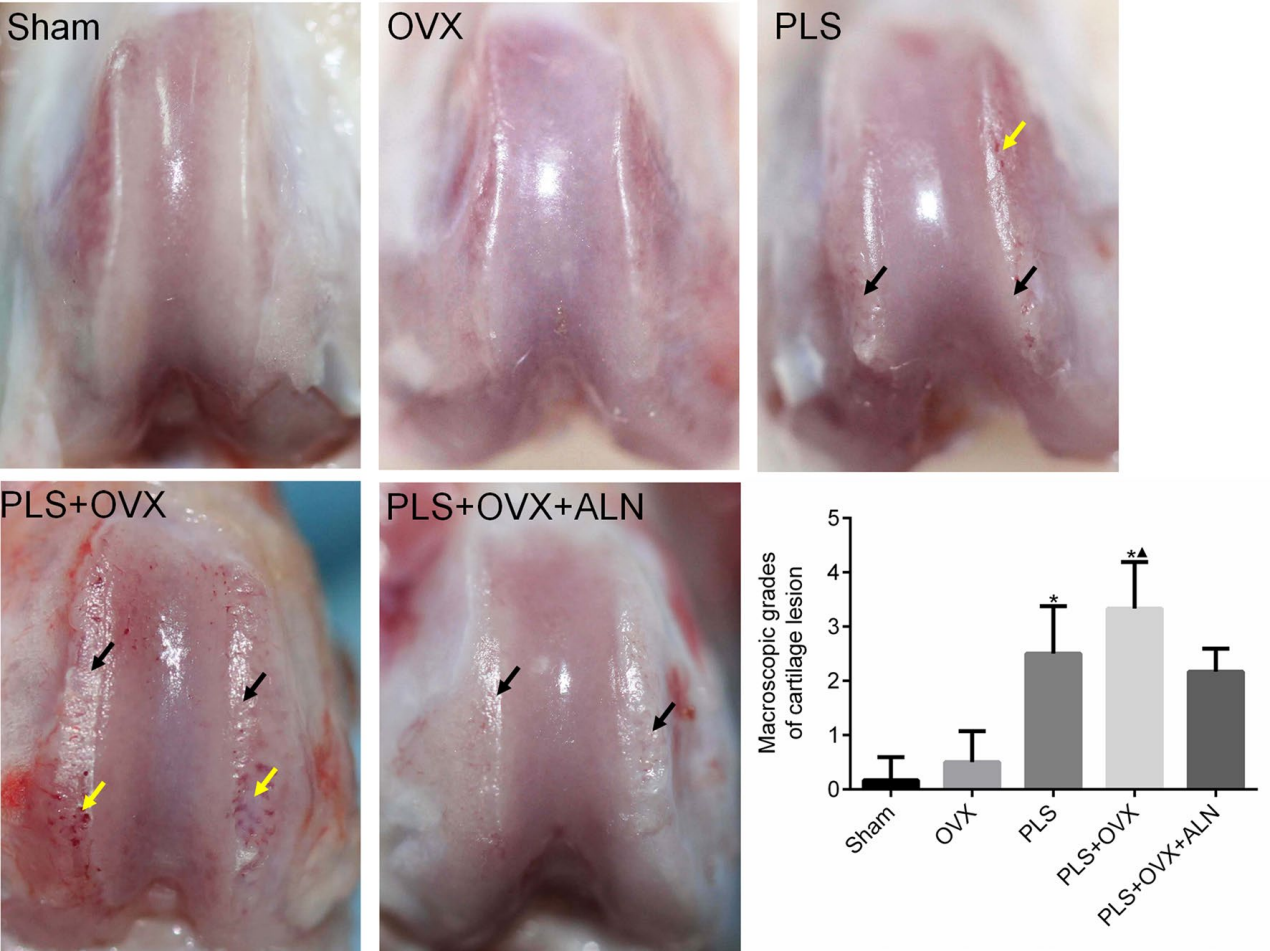
The gross observation of femoral trochlear cartilage degeneration. *p < 0.05 versus sham group, ▲*p* < 0.05 versus OVX group

The gross observation of femoral trochlear cartilage degeneration showed that the Sham group showed normal cartilage appearance with smooth cartilage surface, no color change and no cartilage erosion. In the OVX group, the cartilage color was slightly yellow in some specimens, and the rest showed normal appearance of cartilage. No cartilage erosion was observed. In the PLS group, rough articular cartilage surface was observed, and obvious cartilage erosion appeared in the weight-bearing area of the femoral trochlea, and the injury depth even reached the subchondral bone level. In the PLS + OVX group, severe erosion of the cartilage surface was observed, accompanied by extensive exposure of subchondral bone. Cartilage surface erosion and subchondral bone exposure were improved in PLS + OVX + ALN group compared with PLS + OVX group (Fig. 3).

The gross morphological scores of cartilage degeneration in PLS and PLS + OVX groups were significantly higher than those in Sham group (all *P* < 0.05), and those in PLS + OVX group were significantly higher than those in OVX group (*P* < 0.05). Compared with PLS group, the gross morphological score of PLS + OVX group was increased (*P* > 0.05). Compared with the PLS + OVX group, the gross morphological score of the PLS + OVX + ALN group was decreased (*P* > 0.05) (Fig. 3).

### Histomorphometry

**Fig. 4:**
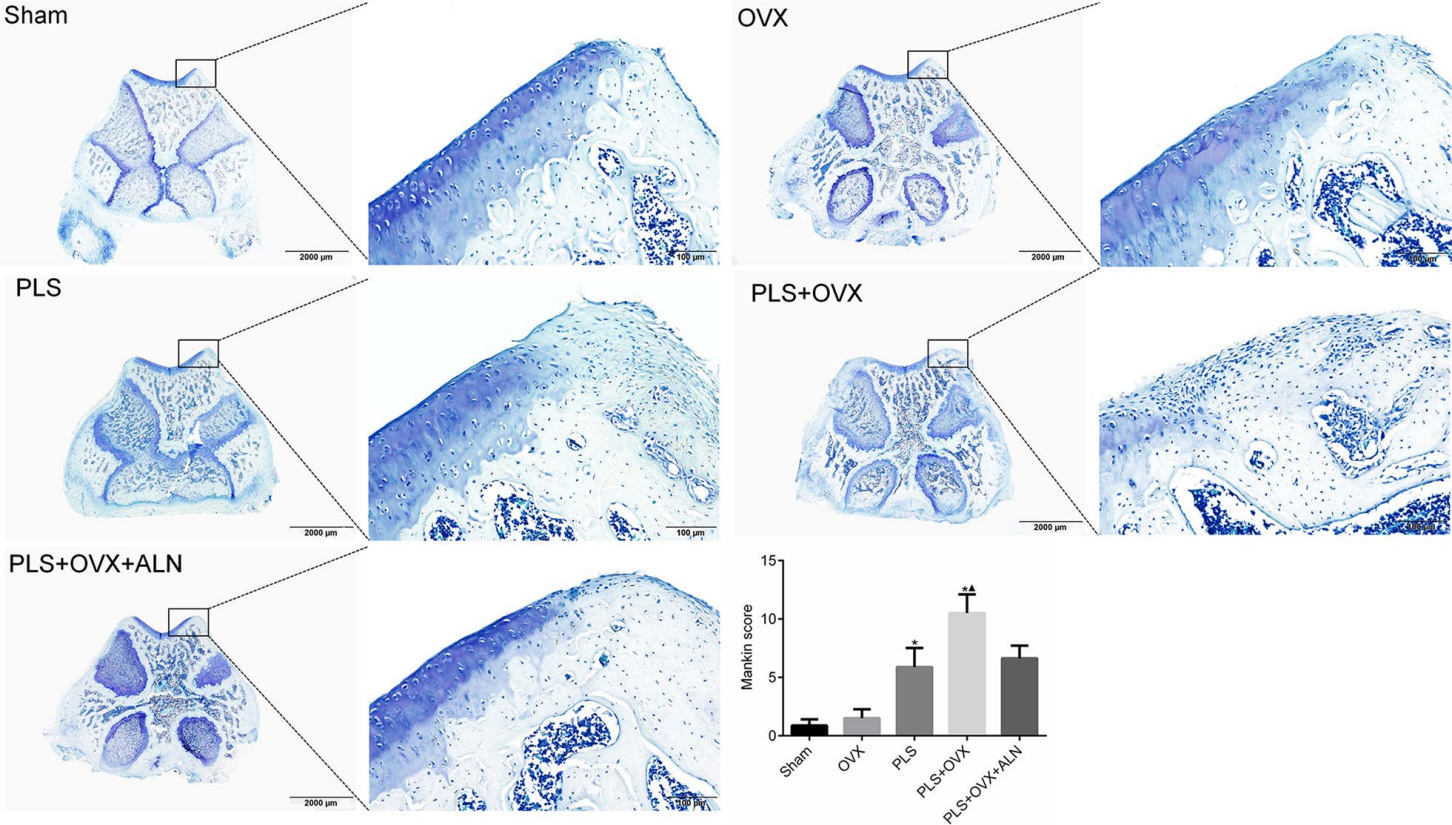
Histomorphology related evaluation. *p < 0.05 versus sham group, ▲*p* < 0.05 versus OVX group

The results of cartilage toluidine blue staining showed that the surface of articular cartilage in Sham group was smooth and complete. No obvious defects were observed. The morphology and distribution of chondrocytes were normal. The matrix staining was uniform, and the tide line was clear and continuous. The OVX group showed a slight decrease in matrix staining intensity, accompanied by an increase in surface chondrocytes. The PLS group showed obvious cartilage degeneration, especially in the load-bearing area of the trochlea, and decreased number of chondrocytes. In the PLS + OVX group, extensive cartilage injury, mass loss of cartilage matrix, obvious fractures and exfoliation of cartilage were observed, and the number of chondrocytes decreased significantly. In PLS + OVX + ALN group, the cartilage surface was slightly damaged, the matrix staining was slightly decreased, the number of chondrocytes was slightly decreased, and the chondrocyte cluster were observed (Fig. 4).

The Mankin score in PLS group and PLS + OVX group was significantly higher than that in Sham group (all *P* < 0.05), and that in PLS + OVX group was significantly higher than that in OVX group (*P* < 0.05). Compared with PLS group, Mankin score in PLS + OVX group was slightly increased (*P* > 0.05). Compared with PLS + OVX + ALN group, Mankin score slightly decreased (*P* > 0.05) (Fig. 4).

### Immunohistochemical findings

**Fig. 5:**
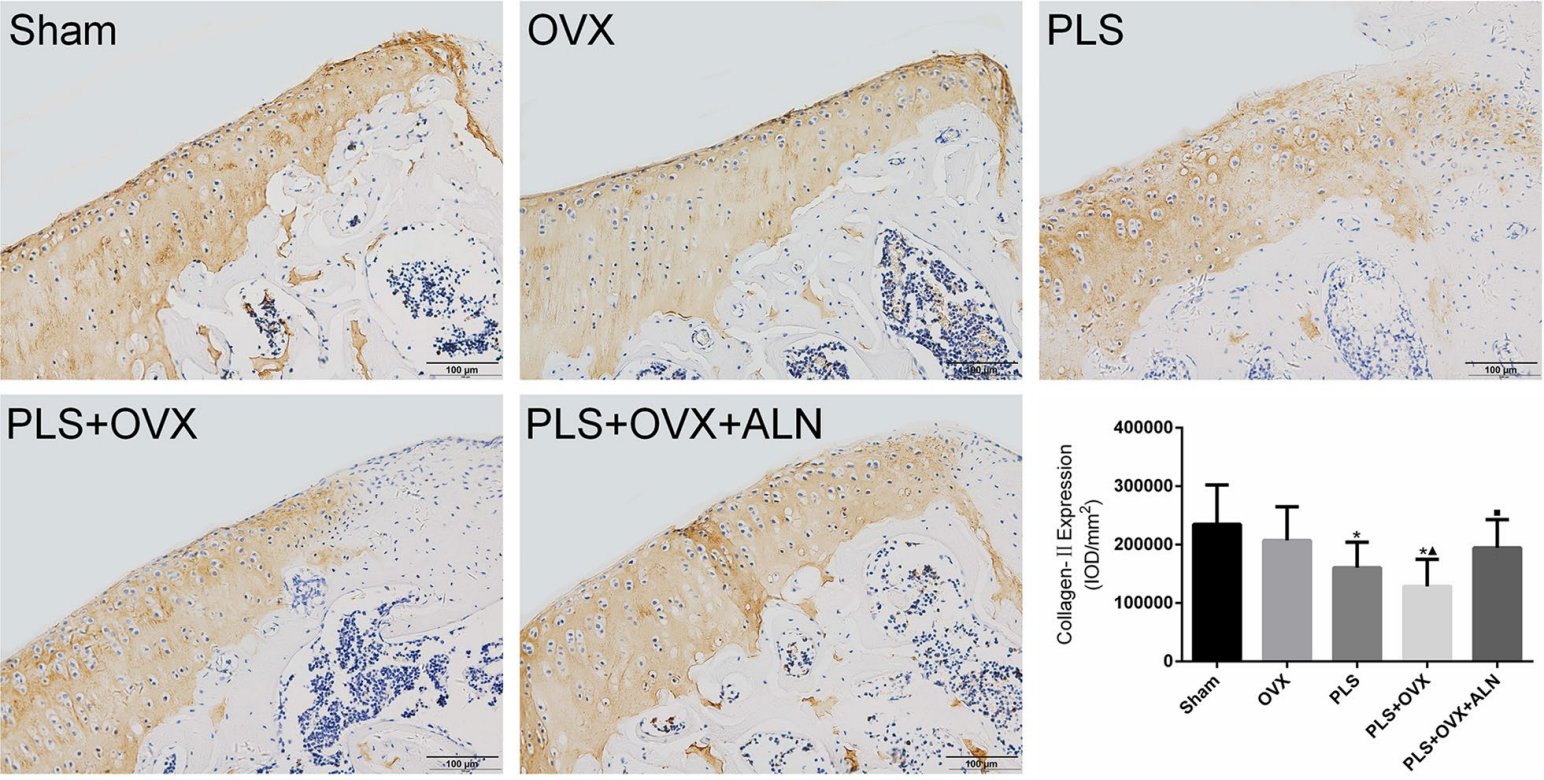
Results of immunohistochemisty staining of Col-II. *p < 0.05 versus sham group, ▲*p* < 0.05 versus OVX group, ■*p* < 0.05 versus PLS + OVX group

The positive expression of Col-II in the cartilage of Sham group was evenly distributed. Compared with Sham group, the expression level of Col-II in PLS group and PLS + OVX group was significantly decreased (all *P* < 0.05), and the expression of Col-II in OVX group was slightly decreased (*P* > 0.05). The expression of Col-II in PLS + OVX group was significantly lower than that in OVX group (*P* < 0.05), and was slightly decreased compared with that in PLS group (*P* > 0.05). The expression level of Col-II in PLS + OVX + ALN group was significantly increased compared with that in PLS + OVX group (*P* < 0.05) (Fig. 5).

**Fig. 6:**
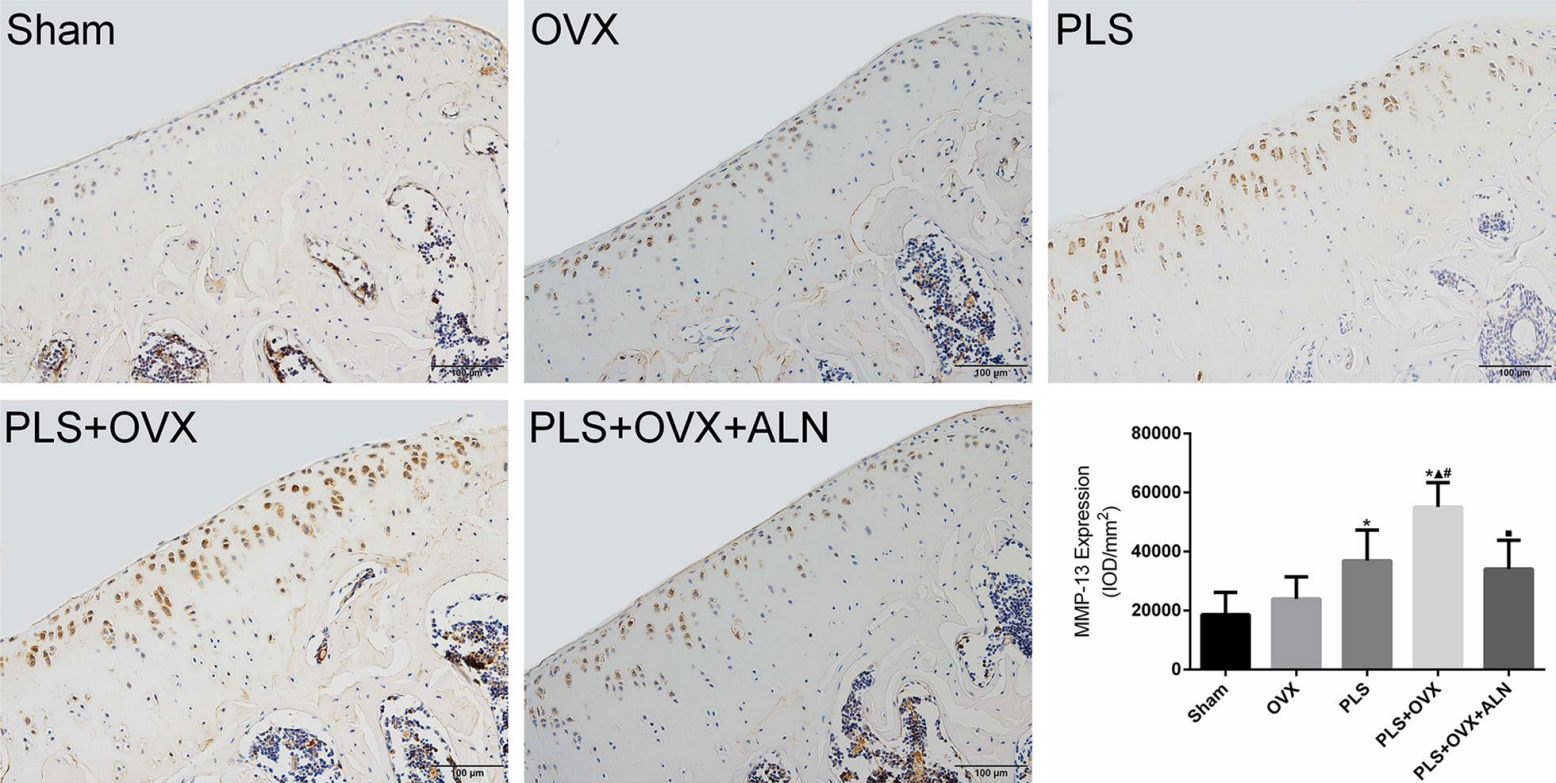
Results of immunohistochemisty staining of MMP-13. *p < 0.05 versus sham group, ▲*p* < 0.05 versus OVX group, #*p* < 0.05 versus PLS group, ■*p* < 0.05 versus PLS + OVX group

MMP-13 is a major collagenase in the pathogenesis of OA, and its expression is increased in the diseased cartilage. The results showed that the positive expression level of MMP-13 in the Sham group was very low. Compared with the Sham group, the expression level of MMP-13 in the PLS group and the PLS + OVX group was significantly increased (all *P* < 0.05), and the expression level of MMP-13 in OVX group was slightly increased (*P* > 0.05). The expression level of MMP-13 in PLS + OVX group was significantly higher than that in OVX group and PLS group (all *P* < 0.05). Compared with PLS + OVX group, the expression level in PLS + OVX + ALN group was significantly decreased (*P* < 0.05) (Fig. 6).

**Fig. 7:**
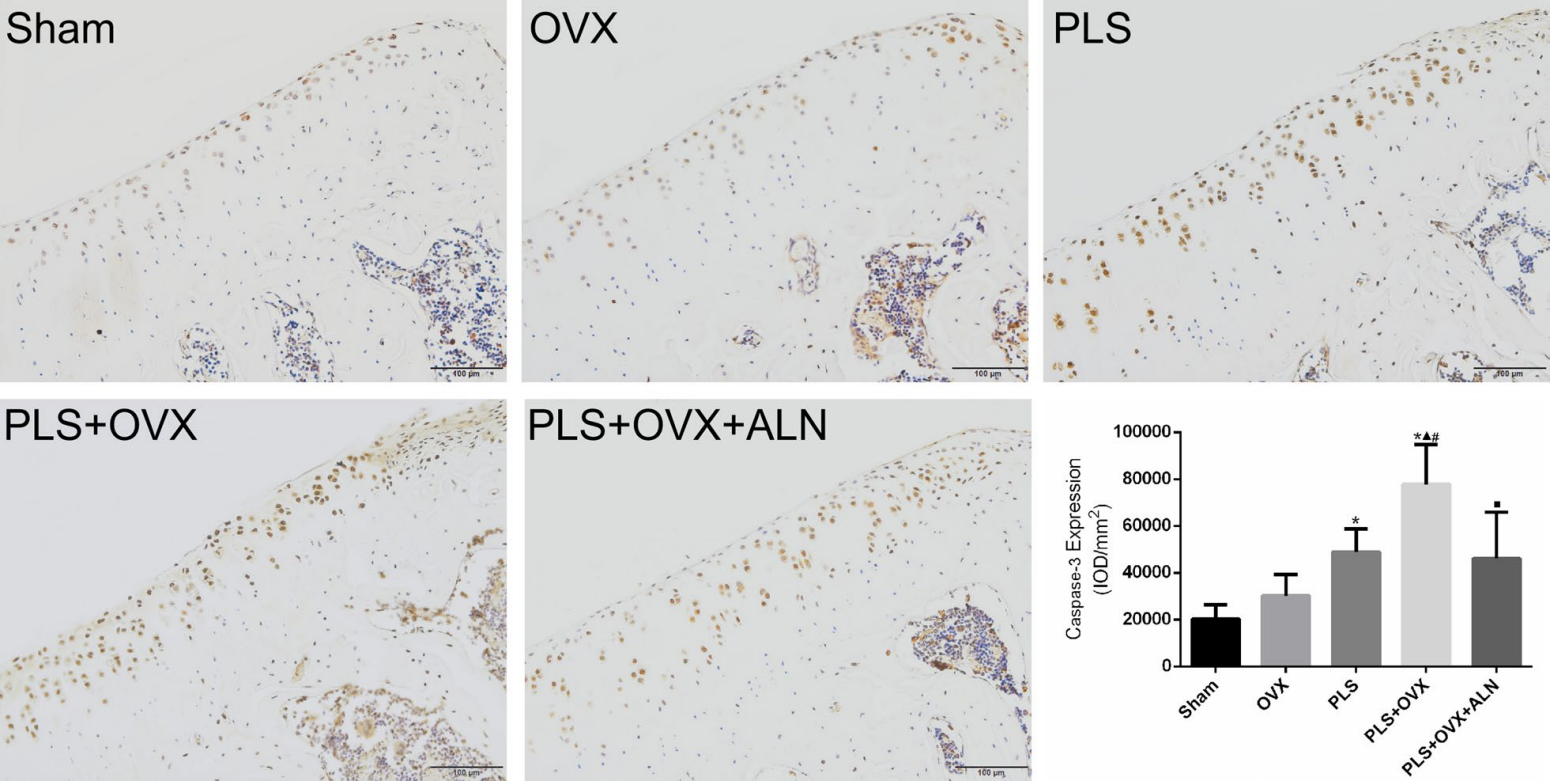
Results of immunohistochemisty staining of Caspase-3. *p < 0.05 versus sham group, ▲*p* < 0.05 versus OVX group, #*p* < 0.05 versus PLS group, ■*p* < 0.05 versus PLS + OVX group

Caspase-3 is a major apoptotic protein in the pathogenesis of OA, and its expression is increased in diseased cartilage. The results showed that the positive expression level of Caspase-3 in the Sham group was very low. Compared with the Sham group, the expression level of Caspase-3 in the PLS group and PLS + OVX group was significantly increased (all *P* < 0.05), and the expression of Caspase-3 in the OVX group was slightly increased (*P* > 0.05). The expression level of Casepase-3 in the PLS + OVX group was significantly higher than that both in the OVX group and PLS group (all *P* < 0.05). Compared with the PLS + OVX group, the expression level in PLS + OVX group was significantly decreased (*P* < 0.05) (Fig. 7).

## Discussion

To our knowledge, this study is the first to investigate the effect of ALN on the pathological process of PFJOA in the OVX rat model. Our results found that early application of ALN can delay the degenerative microstructure changes of articular cartilage and subchondral bone in castrated PFJOA rat to a certain extent, improve and maintain the micro-metabolism and structural changes of cartilage and subchondral bone..

In a broad sense, knee OA is divided into tibiofemoral OA and PFJOA, of which tibiofemoral OA accounts for a large part [19]. Tibiofemoral OA often develops along with TFJOA, and isolated PFJOA is not rare [20]. In the investigation conducted by Noble et al. [21], PFJOA was found in 79% of the 100 cadaver samples that were ≥ 65 years old in their study. The causes of PFJOA include anatomic and mechanical anomalies that can increase local pressure of the patella joint, including patellar trajectory abnormalities such as patella baja [22]. Changes in the anatomical structure and force line of PFJ can cause abnormal changes in the patellar motion trajectory, which not only reduces the contact area between patella and femoral trochlea, but also increases the local stress on the patellofemoral joint surface, resulting in some changes in the uneven forces on both sides of PFJ. Among them, the cartilage matrix on the side with increased stress will wear. The destruction of the original structure of the collagen fiber network and the loss of proteoglycan significantly promote cartilage injury, and finally induce PFJOA, causing anterior knee pain [23, 24].

There are currently many animal models of OA, and how to choose the best model in experimental studies is crucial. However, there is no single gold standard of OA animal model that accurately reflects all aspects of human OA [25]. After the onset of osteoporosis, the change of estrogen level can lead to the change of subchondral bone mass, which affects the stability of the knee joint, and causes the occurrence and development of OA. There are estrogen receptors on the surface of knee cartilage, and the decrease of estrogen level can directly change articular cartilage metabolism and cause OA [26]. In addition, in postmenopausal women, the decline in estrogen levels can directly lead to apoptosis of chondrocytes. Holland et al. [27] confirmed that OA manifestations can occur in the knee joint of sheep after castration surgery. There is a close relationship between the occurrence and development of OA and the decrease of estrogen level [26]. On the preparation of osteoporotic OA animal model by castration, it was found that osteoporosis accelerated the reduction of bone mass in subchondral bone and increased cartilage damage, which proved that osteoporosis is one of the risk factors for the occurrence and development of OA [28, 29].

In our study, bilateral OVX was used to simulate postmenopausal osteoporosis, and PLS was applied to prepare a patella baja model to induce PFJOA, so as to study the effect of decreased estrogen level on PFJOA and the effect of ALN on joint degeneration in ovariectomized rats with PFJOA. Macro and micro analysis showed that PLS and OVX significantly increased the metabolism change and microstructure destruction of cartilage and subchondral bone. The potential mechanism of PLS leading to joint degeneration may be due to the change of movement trajectory and stress increase between patella and femoral trochlea, whose abnormal loads lead to PFJOA-related structural damage.

The occurrence and development of OA is usually accompanied by cartilage injury and abnormal changes of subchondral bone. In the investigation of human OA, partial or full-layer defects of articular cartilage were found in patellofemoral joints during surgery and autopsy [30, 31]. Compared with femoral trochlear cartilage, the fibrosis, longitudinal fissure and softening swelling of human patella cartilage in the early stage are more obvious and serious [32].

Under the condition of OA, chondrocytes will undergo corresponding changes in activity signals. After degeneration, chondrocytes are easy to be induced, differentiated, and proliferated into hypertrophic chondrocytes, resulting in corresponding changes in the biological characteristics of cartilage. These changes in structure and integrity may lead to cartilage injury under the action of stress [33]. Under normal conditions, chondrocytes regulate the production and metabolism of type II collagen, hyaluronic acid and proteoglycan, thus maintaining the normal structure and biomechanical properties of cartilage. In the process of OA, abnormal synthesis and catabolism of these substances often occur in cartilage matrix [34]. These abnormalities can cause the release of inflammatory factors produced by immune cells, including matrix metalloproteinases (MMPs), TNF-α, IL-1β and IL-22, etc. These inflammatory factors affect the normal physiological function of chondrocytes and aggravate cartilage injury [35]. The specific expression of MMP-13 in inflammatory tissues is closely related to its ability to degrade collagen. These proteases degrade collagen, proteoglycan and cartilage oligomeric matrix proteins and affect the normal physiological function of cartilage [36]. These abnormalities in synthesis and catabolism may be related to the decreased level of autophagy in chondrocytes [36]. The decrease of autophagy level of chondrocytes will lead to mitochondrial dysfunction and thus increase apoptosis. Caspase-3 plays a very important or even irreplaceable role in apoptosis. Metabolic abnormalities and oxidative stress caused by mitochondrial dysfunction accelerate the process of chondrocyte injury and cartilage degeneration. Cinque et al. [37] found that the level of autophagy in chondrocytes regulates the synthesis and secretion of type II collagen, which further regulates the changes in the normal physiological structure of cartilage matrix. Similarly, Cheng et al. [38] also found in their study on rabbit OA model that the increase of chondrocyte autophagy level was closely related to the degenerative changes of articular cartilage, and involved in the occurrence and development of OA.

There is a close relationship between SB and cartilage in anatomical structure, and the two have mutual influence and correlation in biological, biomechanical and pathological changes. SB is located below the cartilage and is composed of the SB plate and the underlying cancellous bone, which supports and cushions the stress of the cartilage [28]. The SB plate is a thin cortical layer with pores, which provides the structural basis for the communication between articular cartilage and SB. Iijima et al. [39] found in their study on the animal model of OA that the expressions of MMP-13 and VEGF in SB would increase with the progression of OA, while the fibrosis of SB defects was significant. In the development of OA, the balance between osteoblasts and osteoclasts determines the formation or degradation of bone matrix during bone reconstruction. In the early stage of OA, bone resorption and bone remodeling are the main processes, the bone metabolic rate of the remodeling site increases, and the bone plate thickness decreases [40]. While late OA is characterized by decreased bone resorption and increased bone formation. Cheng et al. [41] found that the bone density of SB decreased significantly in the early stage of OA, but with the progression of the disease, the bone density of SB gradually increased, and the SB became sclerotic. After the bone trabeculae were damaged, the stress resistance of articular cartilage and SB declined. Bellido et al. [42] found on the pig model that in the process of OA, SB changes before cartilage. When the elastic modulus of SB decreases, the stress borne by articular cartilage increases significantly, resulting in degenerative changes of cartilage.

In our study, both PLS and OVX accelerated cartilage and SB degeneration. PLS and OVX increased the expression of catabolic enzyme MMP-13 and apoptotic protein caspase-3, and promoted the degradation of cartilage microstructure and the enhancement of catabolism of Col II. Micro-CT and histomorphologic analysis revealed bone loss and microstructure deterioration of SB.

Due to its role in regulating bone metabolism and anti-bone resorption, ALN is mainly used in the treatment of osteoporosis in clinical practice, especially for postmenopausal osteoporosis [43]. Early prophylactic treatment with sodium alendronate in the course of OA disease can reduce the metabolic rate of SB and alleviate the degeneration of articular cartilage, while late administration has no significant effect on articular cartilage, indicating that early treatment to protect SB has a positive role in preventing cartilage destruction [44].

ALN can improve the abnormal changes of SB. Hayami T et al. prepared a rat OA model through ACLT surgery and observed that ALN intervention had a significant inhibitory effect on the absorption of knee SB in OA rats [45]. M. Siebelt et al. [46] studied the inhibitory effect of ALN on osteoclast bone absorption and found that ALN treatment after OA induction could reduce SB loss and osteophyte formation compared with rats in the non-ALN treatment group. ALN treatment also improved the degree of cartilage degradation. In the study on the effect of ALN on OA, Zhang et al. [47] found that compared with the modeling group, the related SB morphometric analysis indexes, such as BMD, Tb.N, BV/TV, Tb.Th, Tb.Sp, Tbpf and SMI, in the ALN group were significantly improved.

MMP-13 has a direct degradation effect on Col-II, and its changes can directly reflect the changes in the metabolism level of Col-II in cartilage matrix. Shirai et al. [48] established a rabbit OA model by ACLT and intervened with subcutaneous injection of ALN. The experimental results showed that ALN could prevent the loss of SB mass. Obvious cartilage damage occurred in the model group, while only slight cartilage destruction was observed in the ALN group. It was observed that ALN inhibited the expression of MMP-13 in cartilage, and the application of ALN could improve the local metabolism of articular cartilage, thereby preventing the degeneration of articular cartilage. Zhang et al. [47] found that the application of ALN can increase the expression of bone morphogenetic protein (BMP-2) and inhibit the expression of MMP-13, which can effectively inhibit cartilage degradation and thus exert its protective effect on cartilage. Chen et al. [49] found that early ALN treatment could completely inhibit cartilage thickening and effectively improve SB degeneration in OA rats, while late ALN treatment had no effect. ALN not only has a protective effect on articular cartilage and SB, but also has been confirmed to prevent the formation of osteophytes [40, 47].

Our study found that early application of ALN has a protective effect on cartilage and SB. ALN inhibited the expression of MMP-13 and Caspase-3 in cartilage, so reduced the catabolism of cartilage and apoptosis of chondrocytes, and played a protective role in articular cartilage. Early application of ALN can inhibit bone turnover and bone loss in SB and improve the microstructure of SB. Articular cartilage and SB complement each other and influence each other functionally. The protective effect of ALN on articular cartilage is the result of a comprehensive effect. The protective effect of ALN on articular cartilage may be related to the inhibition of SB bone remodeling, or may be the result of its direct action on cartilage.

Khorasani et al. [50] study on the effect of ALN on post-traumatic OA induced by anterior cruciate ligament rupture in mice. They found that high-dose ALN treatment prevented early trabecular bone loss and cartilage degeneration after non-invasive knee injury, but did not mitigate long-term joint degeneration. Therefore, these data contribute to the understanding of the effects of bisphosphonates on the development of OA and may support the early use of anti-absorption drugs to prevent joint degeneration after injury, although further research is needed.

## Conclusion

PLS can successfully produce animal models of PFJOA caused by patella baja, resulting in degeneration of cartilage and subchondral bone. OVX caused a decrease in estrogen levels in rats, which aggravated the joint degeneration caused by PFJOA. The application of ALN can delay the destruction of articular cartilage and subchondral bone microstructure in castrated PFJOA rat to a certain extent.

### Supplementary Information

**Additional file 1**. The surgical procedure of patellar ligaments shorting (PLS) surgery (A–B): A 1 cm longitudinal incision was made medially along the patella to the tibial tuberosity followed by shaving and sterilizing the skin of the right knee. (C–D): The patellar tendon was separated by hemostatic forceps and 1-0 Vicryl sutures without exposing the joint cavity. (E): a Kirschner wire (7 mm long, 2 mm diameter) with 1-0 nylon sutures in a groove 1 mm medial to both ends was inserted under the patellar tendon from the medial to the lateral region. (F): Sutures crossed at the proximal end of the tendon. (G-H): sutures were threaded under both grooves and tightly tied around the patellar tendon with the knee in its straightest position; (I) skin was sealed using 3-0 nylon sutures.

## Data Availability

The datasets generated during and/or analyzed during the current study are publicly available.
